# The VEGF-A inhibitor sFLT-1 improves renal function by reducing endothelial activation and inflammation in a mouse model of type 1 diabetes

**DOI:** 10.1007/s00125-017-4322-3

**Published:** 2017-06-15

**Authors:** Pascal Bus, Marion Scharpfenecker, Priscilla Van Der Wilk, Ron Wolterbeek, Jan A. Bruijn, Hans J. Baelde

**Affiliations:** 1 Department of Pathology, Leiden University Medical Center, L1Q, Room P0-107, P.O. Box 9600, 2300 RC Leiden, the Netherlands; 2 Department of Medical Statistics and Bioinformatics, Leiden University Medical Center, Leiden, the Netherlands

**Keywords:** Albuminuria, Diabetic nephropathy, Endothelial activation, Glomerular damage, Inflammation, Renal function, sFLT-1, VEGF-A

## Abstract

**Aims/hypothesis:**

Animal models of diabetic nephropathy show increased levels of glomerular vascular endothelial growth factor (VEGF)-A, and several studies have shown that inhibiting VEGF-A in animal models of diabetes can prevent albuminuria and glomerular hypertrophy. However, in those studies, treatment was initiated before the onset of kidney damage. Therefore, the aim of this study was to investigate whether transfecting mice with the VEGF-A inhibitor *sFlt-1* (encoding soluble fms-related tyrosine kinase 1) can reverse pre-existing kidney damage in a mouse model of type 1 diabetes. In addition, we investigated whether transfection with *sFlt-1* can reduce endothelial activation and inflammation in these mice.

**Methods:**

Subgroups of untreated 8-week-old female C57BL/6J control (*n* = 5) and diabetic mice (*n* = 7) were euthanised 5 weeks after the start of the experiment in order to determine the degree of kidney damage prior to treatment with sFLT-1. Diabetes was induced with three i.p. injections of streptozotocin (75 mg/kg) administered at 2 day intervals. Diabetic nephropathy was then investigated in diabetic mice transfected with *sFlt-1* (*n* = 6); non-diabetic, non-transfected control mice (*n* = 5); non-diabetic control mice transfected with *sFlt-1*(*n* = 10); and non-transfected diabetic mice (*n* = 6). These mice were euthanised at the end of week 15. Transfection with *sFlt-1* was performed in week 6.

**Results:**

We found that transfection with *sFlt-1* significantly reduced kidney damage by normalising albuminuria, glomerular hypertrophy and mesangial matrix content (i.e. glomerular collagen type IV protein levels) (*p* < 0.001). We also found that transfection with *sFlt-1* reduced endothelial activation (*p* < 0.001), glomerular macrophage infiltration (*p* < 0.001) and glomerular TNF-α protein levels (*p* < 0.001). Finally, sFLT-1 decreased VEGF-A-induced endothelial activation in vitro (*p* < 0.001).

**Conclusions/interpretation:**

These results suggest that sFLT-1 might be beneficial in treating diabetic nephropathy by inhibiting VEGF-A, thereby reducing endothelial activation and glomerular inflammation, and ultimately reversing kidney damage.

**Electronic supplementary material:**

The online version of this article (doi:10.1007/s00125-017-4322-3) contains peer-reviewed but unedited supplementary material, which is available to authorised users.

## Introduction

Diabetic nephropathy is characterised by damage and dysfunction of the microvasculature [1]. A critical factor in maintaining the microvasculature is vascular endothelial growth factor (VEGF)-A, which regulates many aspects of vascular physiology, including vascular permeability and the migration, proliferation and survival of endothelial cells (for review, see Bartlett et al [2]). Several studies in both human and animal models have indicated that proper glomerular function requires tight regulation of VEGF-A levels, as both upregulation and downregulation of VEGF-A can lead to kidney disease [3].

Animal models of diabetic nephropathy develop increased levels of glomerular VEGF-A [4, 5], possibly due to the effect of high glucose on VEGF-A production in podocytes [6]. Therefore, inhibiting VEGF-A may be beneficial in treating renal complications. Consistent with this notion, antibodies directed against VEGF-A have been shown to prevent albuminuria [7, 8] and glomerular hypertrophy [9] in animal models of diabetes. However, in these studies, the inhibition of VEGF-A was initiated prior to the onset of diabetic kidney disease (i.e. prior to the development of albuminuria, glomerular hypertrophy and mesangial expansion/matrix production); thus, whether this strategy is feasible for treating diabetic people with existing kidney damage is currently unknown.

In addition to its role in maintaining vascular homeostasis, VEGF-A also facilitates the migration of monocytes and macrophages. Several studies have found that macrophages play a role in diabetic nephropathy [10–12]. VEGF-A-induced migration of monocytes and macrophages is mediated by the binding of VEGF-A to VEGF receptor (VEGFR)-1 (also known as fms-related tyrosine kinase (FLT)-1) expressed on these cells [13–15]. In addition, both VEGF-A [16] and high glucose levels [17] can activate endothelial cells, leading to increased levels of vascular cell adhesion molecule (VCAM)-1 and intercellular adhesion molecule (ICAM)-1, thereby promoting monocyte infiltration.

Here, we investigated whether the VEGF-A inhibitor soluble FLT-1 (sFLT-1; also known as soluble VEGFR-1) can reduce renal complications, including albuminuria and mesangial matrix expansion, in a mouse model of type 1 diabetes and pre-existing kidney damage. In addition, because diabetic nephropathy is accompanied by endothelial activation [1] and macrophage infiltration [11, 12, 18], both of which are mediated by VEGF-A, we also investigated the effect of inhibiting VEGF-A on these variables. Last, we investigated whether transfection with *sFlt-1* reduces glomerular TNF-α protein levels (a measure of inflammation) in diabetic mice.

## Methods

### *sFlt-1* transfection

pcDNA3.1 vectors (Invitrogen, Breda, the Netherlands) containing either mouse *sFlt-1-VSV* or the luciferase gene, both of which are driven by the cytomegalovirus promoter, were constructed as described previously [19]. The plasmids were amplified in *Escherichia coli* DH5α (Invitrogen), purified using the QIAfilter Plasmid Maxi-prep kit (Qiagen, Venlo, the Netherlands) and dissolved in EndoFree Tris–EDTA buffer (Qiagen). The mice were co-transfected with the *sFlt-1-VSV* and luciferase constructs in both calf muscles (20 μg each) using electroporation, as described previously [19]. To monitor transfection efficiency, the mice were injected with i.p. luciferin at 2-week intervals. Five minutes after the luciferin injection, luciferase activity was visualised using a NightOWL bioluminescence camera (Xenogen Ivis Spectrum, Alameda, CA, USA), as described previously [19].

### Tube formation assay

To confirm functional expression of the *sFlt-1* construct, we performed a tube formation assay as described previously [20]. In brief, human umbilical vein endothelial cells (HUVECs) (1.5 × 10^3^ cells per well; Promocell, Heidelberg, Germany) were plated on Matrigel-coated 96-well plates (Corning, Amsterdam, the Netherlands). The HUVECs were incubated for 6 h with culture medium obtained from human embryonic kidney 293 (HEK293) cells (ATCC, Manassas, VA, USA) transfected with an *sFlt-1* construct (2 μg) or a luciferase construct (2 μg). The HEK293 cells were transfected using 6 μl X-tremeGENE (Roche, Basel, Switzerland); 2 days after transfection, the culture medium was collected and applied to the HUVECs in the presence or absence of VEGF-A (10 ng/ml; R&D Systems, Minneapolis, MN, USA). The number of tube branch points was counted in five ×400 fields. Images were taken using a Moticam camera (Motic, Xiamen, China). This experiment was performed three times.

### Animals

This study used 8-week-old female C57BL/6J mice (specific pathogen free; Harlan Laboratories, Indianapolis, IN, USA), weighing 17.8 ± 1.1 g (mean ± SD). All experiments were conducted in accordance with national guidelines for the care and use of experimental animals (DEC license 13163). Mice were housed in individually ventilated cages in groups of five mice, with food and water ad libitum. C57BL/6J mice were chosen because this study was a follow-up of a previous study that investigated podocyte-specific VEGF-A knockdown on a C57BL/6 background [21]. Moreover, C57BL/6J mice respond well to the streptozotocin (STZ) regimen in terms of blood glucose levels [22].

Diabetes was induced with three i.p. injections of STZ (75 mg/kg body weight; Sigma-Aldrich, St Louis, MO, USA) administered at 2 day intervals. Blood glucose levels were measured (Accu-Chek; Roche) at the end of weeks 1, 5 and 15 after diabetes induction. Mice with a blood glucose level of 15 mmol/l or higher were considered diabetic. Mice were randomly divided into groups. Subgroups of untreated control mice (*n* = 5) and diabetic mice (*n* = 10) were killed 5 weeks after the start of the experiment in order to determine the degree of kidney damage prior to treatment with sFLT-1. In week 6, the mice were transfected with a plasmid containing *sFlt-1*. Diabetic nephropathy was then investigated in diabetic mice transfected with *sFlt-1* (*n* = 10), non-diabetic, non-transfected control mice (*n* = 5), non-diabetic control mice transfected with *sFlt-1* (*n* = 10) and non-transfected diabetic mice (*n* = 10). These mice were killed at the end of week 15. Three diabetic mice 5 weeks after the induction of diabetes, four diabetic mice transfected with *sFlt-1* and four diabetic mice 15 weeks after the induction of diabetes were excluded from the study as they did not meet the inclusion criteria of a blood glucose level of 15 mmol/l or higher.

### Measurement of the urine albumin excretion ratio

To measure the urine albumin excretion ratio, spot urine was collected in weeks 5 and 15. Urine albumin levels were measured using rocket immunoelectrophoresis with rabbit anti-mouse albumin; purified mouse serum albumin (Sigma-Aldrich) was used as a standard. Urine creatinine was measured using a creatinine assay, with picric acid, sodium hydroxide and creatinine standards (Sigma-Aldrich); the albumin:creatinine ratio was then calculated.

### Immunohistochemistry

Paraffin-embedded kidney tissues (4 μm thickness) were cut using a Leica microtome (Wetzlar, Germany) and stained with periodic acid–Schiff’s reagent using a standard protocol. Rabbit anti-mouse platelet/endothelial cell adhesion molecule 1 (PECAM-1; 1:400; Santa Cruz Biotechnology, Dallas, TX, USA), rabbit anti-human Wilms tumour (WT)1 (1:500; Santa Cruz Biotechnology) and rabbit anti-mouse collagen type IV (1:200; Abcam, Cambridge, UK) primary antibodies were used for immunostaining, followed by the anti-rabbit-Envision HRP-conjugated secondary antibody (undiluted; Dako, Glostrup, Denmark), with diaminobenzidine (DAB+; Dako) as the chromogen. The rabbit anti-human WT1 antibody cross-reacts with mouse WT1 (data not shown). As a negative control, non-specific isotype matched antibodies were used.

Frozen kidney tissues (4 μm thickness) were cut using a Leica cryostat. Rabbit anti-mouse fibronectin (1:2400; Sigma-Aldrich), rat anti-mouse CD68 (1:15; Abcam), rat anti-mouse VCAM-1 (1:1400; BD Pharmingen, San Diego, CA, USA), rat anti-mouse ICAM-1 (1:200; ATCC), rabbit anti-mouse TNF-α (1:100; Abcam) and rabbit anti-vesicular stomatitis virus (VSV; 1:2500; Sigma-Aldrich) primary antibodies were used for immunostaining, followed by the appropriate Envision (undiluted; Dako) or Impress (undiluted; Vector Laboratories, Burlingame, CA) HRP-conjugated secondary antibody, with DAB+ as the chromogen. As a negative control, non-specific isotype matched antibodies were used. Antibodies were tested for specificity with western blot analysis (PECAM-1, WT1, fibronectin, CD68, ICAM-1, TNF-α, VSV), immunoprecipitation (VCAM-1) or immunogen affinity purified (collagen type IV).

### Digital image analysis

Sections were digitised using the Philips Ultra-Fast Scanner 1.6 RA (Amsterdam, the Netherlands). The surface area of the glomerular tuft (in μm^2^) was measured in periodic acid–Schiff’s reagent-stained slides with 25 glomeruli per section using Philips Ultra-Fast Scanner 1.6 RA software (Philips). ImageJ software (https://imagej.nih.gov/ij/) was used to measure the levels of fibronectin, collagen type IV, PECAM-1, VCAM-1, ICAM-1 and TNF-α. The positive area per glomerulus was determined by measuring the respective positively stained area, corrected for the total area of the glomerulus (ten and 25 glomeruli per frozen and paraffin-embedded section, respectively) at ×400 magnification. The number of podocytes in each sample was determined by counting the number of WT1-positive nuclei per glomerulus in 25 glomeruli. The number of macrophages was determined by counting the number of CD68-positive cells in ten glomeruli. The glomeruli used for these measurements were selected at random. Experimenters were blind to group assignment and outcome assessment.

### Endothelial activation assay

HUVECs that were confluent for 2 days were incubated with VEGF-A (20 ng/ml; R&D Systems) for 2, 4, 6 and 8 h. To determine the effect of sFLT-1 on VEGF-A-induced endothelial activation, HUVECs were incubated for 4 h with sFLT-1 (0, 10, 100 or 1000 ng/ml; R&D Systems) in the presence of 20 ng/ml VEGF-A. These experiments were performed three times. Cell lines were negative for mycoplasma contamination.

To quantify changes in gene expression, total RNA was extracted from HUVECs using TRIzol extraction buffer (ThermoFisher Scientific, Waltman, MA, USA) and converted to cDNA with AMV reverse transcriptase (Roche) using random hexamer primers. Quantitative real-time PCR was performed using IQ SYBR Green Supermix (Bio-Rad, Hercules, CA, USA) on a Bio-Rad CFX real-time system. Cycle threshold values were normalised to the housekeeping gene *Hprt1*. The following primers were used in this study: *HPRT1*: 5′-AGATGGTCAAGGTCGCAAGC-3′ and 5′-TCAAGGGCATATCCTACAACAAAC-3′; *ICAM-1*: 5′-CAGAGGTTGAACCCCACAGT-3′ and 5′-CCTCTGGCTTCGTCAGAATC-3′; *SELE*: 5′-AGCCCAGAGCCTTCAGTGTA-3′ and 5′-AACTGGGATTTGCTGTGTCC-3′. Primers for amplifying *VCAM-1* were obtained from Sino Biological (North Wales, PA, USA).

### Statistical analyses

Data are expressed as means ± SD. Data were analysed using the two-tailed Student’s *t* test or one-way ANOVA. We also used a one-way ANOVA to analyse the effect of sFLT-1 treatment in diabetic mice (week 15), corrected for the effect of time. Differences were considered significant at *p* < 0.05.

## Results

### Transfection with *sFlt-1* reduced endothelial tube formation in vitro

**Fig. 1:**
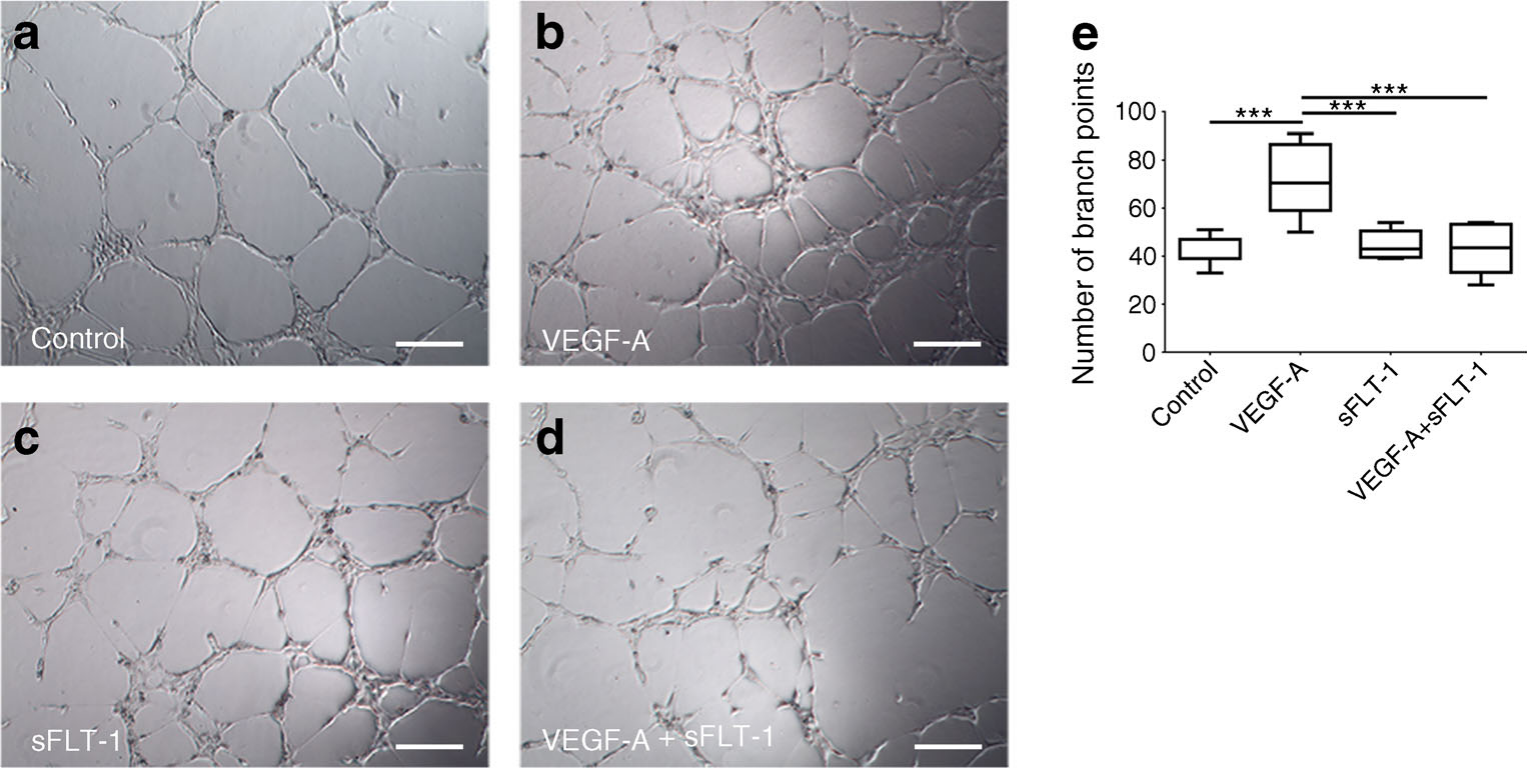
sFLT-1 inhibited VEGF-induced tube formation in vitro. (**a**–**d**) HUVECs were cultured in the presence or absence of VEGF-A (10 ng/ml) and/or sFLT-1, and the number of branch points was measured. (**e**) Summary of the total number of branch points measured in five fields under each condition. Boxes represent 1st and 3rd quartiles; whiskers represent minimum and maximum number of branch points; horizontal line represents median number of branch points. ***p < 0.001, one-way ANOVA. Scale bars, 100 μm

To confirm functional expression of the *sFlt-1* construct, we performed a tube formation assay. First, HUVECs were cultured in medium obtained from luciferase-transfected HEK293 cells. The addition of VEGF-A (10 ng/ml) to the culture medium led to increased tube formation (Fig. 1a, b), reflected by an increased number of branch points (Fig. 1e). VEGF-A-induced tube formation was significantly inhibited by medium obtained from *sFlt-1*-transfected HEK293 cells (Fig. 1d), confirming that expression of the *sFlt-1* construct inhibits VEGF-A-induced tube formation. As a control, culturing HUVECs with medium obtained from *sFlt-1-*transfected HEK293 cells had no effect on tube formation in the absence of VEGF-A (Fig. 1c).

### Expression of sFLT-1 in mice by co-transfection with the *sFlt-1-VSV* and luciferase constructs

Diabetes was induced in mice by i.p. injections of STZ (see Methods). In week 6, mice were transfected with the *sFlt-1-VSV* and luciferase constructs by bilateral injection in the calf muscle. Transfection was confirmed by injecting the mice with luciferin (see electronic supplementary material [ESM] Fig. 1). Staining for VSV was used to confirm the presence of exogenous sFLT-1 in the renal vasculature (data not shown).

### Transfection with *sFlt-1* reduced kidney damage in diabetic mice

**Fig. 2:**
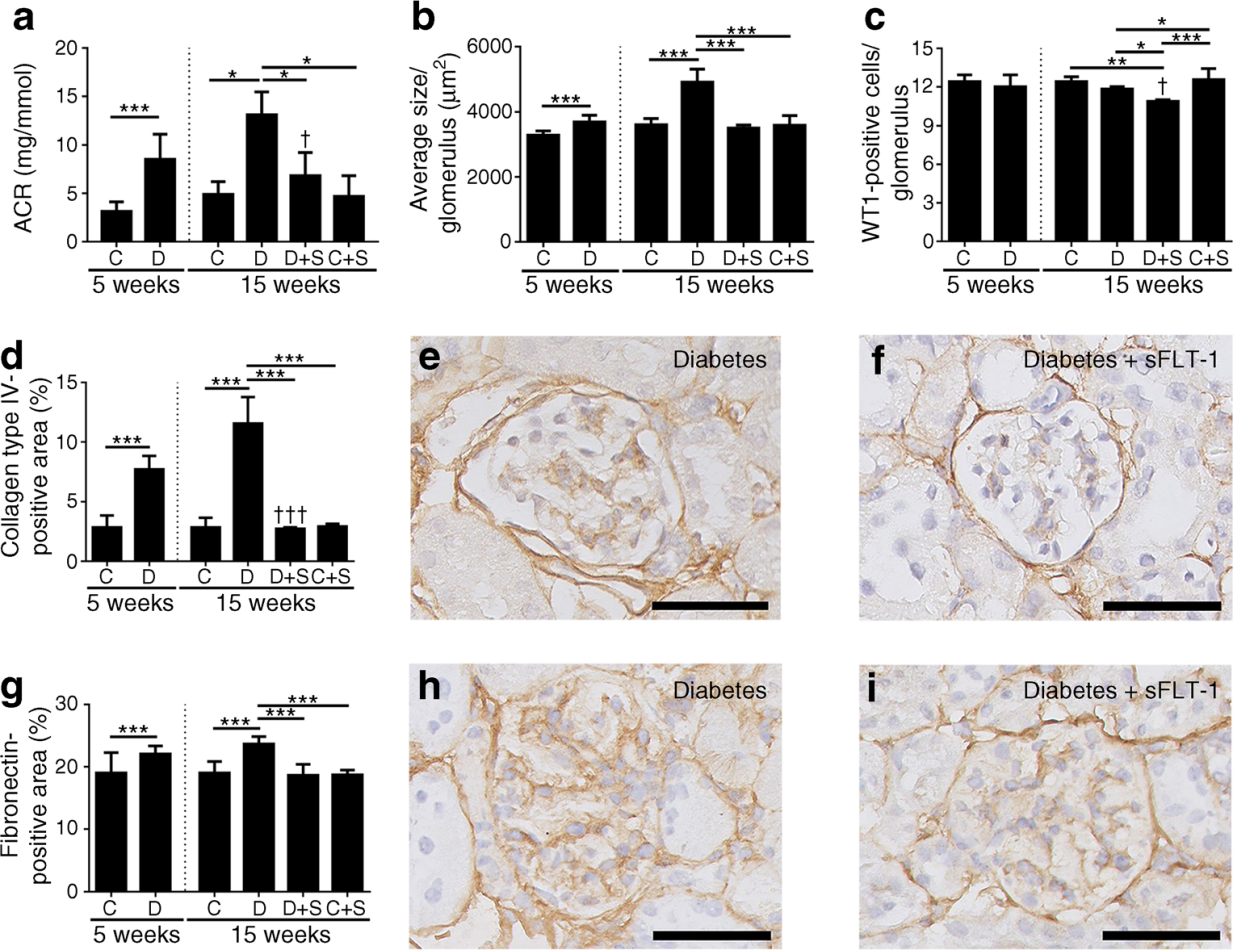
sFLT-1 reversed kidney damage in diabetic mice. Mice were injected with STZ to induce diabetes. In week 6, diabetic (D) and control (C) mice were transfected with a construct expressing *sFlt-1* (S). At 5 and 15 weeks, albuminuria (**a**; albumin:creatinine ratio [ACR]), glomerular hypertrophy (**b**), glomerular podocytes (**c**), collagen type IV positivity (**d**) and fibronectin positivity (**g**) were measured. (**e**, **f**) Representative images of collagen type IV immunostaining in an untreated diabetic mouse at week 15 (**e**) and a diabetic mouse transfected with *sFlt-1* (**f**). (**h**, **i**) Representative images of fibronectin immunostaining in an untreated diabetic mouse at week 15 (**h**) and a diabetic mouse transfected with *sFlt-1* (**i**). ***p < 0.001, Student’s *t* test between groups at week 5. *p < 0.05, **p < 0.01 and ***p < 0.001, one-way ANOVA between groups at week 15. ^†^ *p* < 0.05 and ^†††^ *p* < 0.001 vs the corresponding diabetic mice at 5 weeks, one-way ANOVA after correcting for the time effect. Bars represent means ± SD. Number of animals: non-diabetic, non-transfected control mice at 5 and 15 weeks (*n* = 5 mice each); non-transfected diabetic mice at 5 and 15 weeks (*n* = 7 and *n* = 6, respectively); non-diabetic control mice transfected with *sFlt-1* (*n* = 10); and diabetic mice transfected with *sFlt-1* (*n* = 6). Scale bars, 50 μm

We first determined the development of kidney damage in diabetic mice 5 weeks after diabetes was induced. Inducing diabetes led to albuminuria, reflected by an albumin:creatinine ratio of 8.53 ± 2.59 mg/mmol, which was significantly higher than in control mice (3.06 ± 0.98 μg/mg; *p* < 0.001) (Fig. 2a). In addition, compared with control mice, diabetic mice developed glomerular hypertrophy (*p* < 0.001) (Fig. 2b). Podocyte numbers did not differ between diabetic and control mice (Fig. 2c). The protein levels of both collagen type IV and fibronectin—two markers of mesangial matrix expansion—were higher in the diabetic mice compared with control mice (*p* < 0.001) (Fig. 2d, e, f and Fig. 2g, h, i, respectively).

Having confirmed that kidney damage develops in these mice within 5 weeks, we next examined the effect of *sFlt-1* transfection; transfection with *sFlt-1* was performed in week 6 and the mice were analysed 9 weeks after transfection (i.e. 15 weeks after diabetes was induced). Our analysis revealed that sFLT-1 significantly reduced all markers of kidney damage in the diabetic mice, including albuminuria, glomerular hypertrophy and mesangial matrix expansion (*p* < 0.001) (Fig. 2a, b, d, g). Compared with control-transfected diabetic mice, *sFlt-1*-transfected diabetic mice had significantly fewer podocytes (*p* < 0.01) (Fig. 2c). Transfecting control (i.e. non-diabetic) mice with *sFlt-1* had no effect on any of the markers investigated (Fig. 2). Finally, compared with diabetic mice at week 5, *sFlt-1*-transfected diabetic mice at week 15 had significantly lower levels of albuminuria and collagen type IV (*p* < 0.05 and *p* < 0.001, respectively), indicating that transfection with *sFlt-1* can reverse pre-existing kidney damage (Fig. 2a, d).

### Transfection with *sFlt-1* reduced endothelial activation and inflammation in diabetic mice

**Fig. 3:**
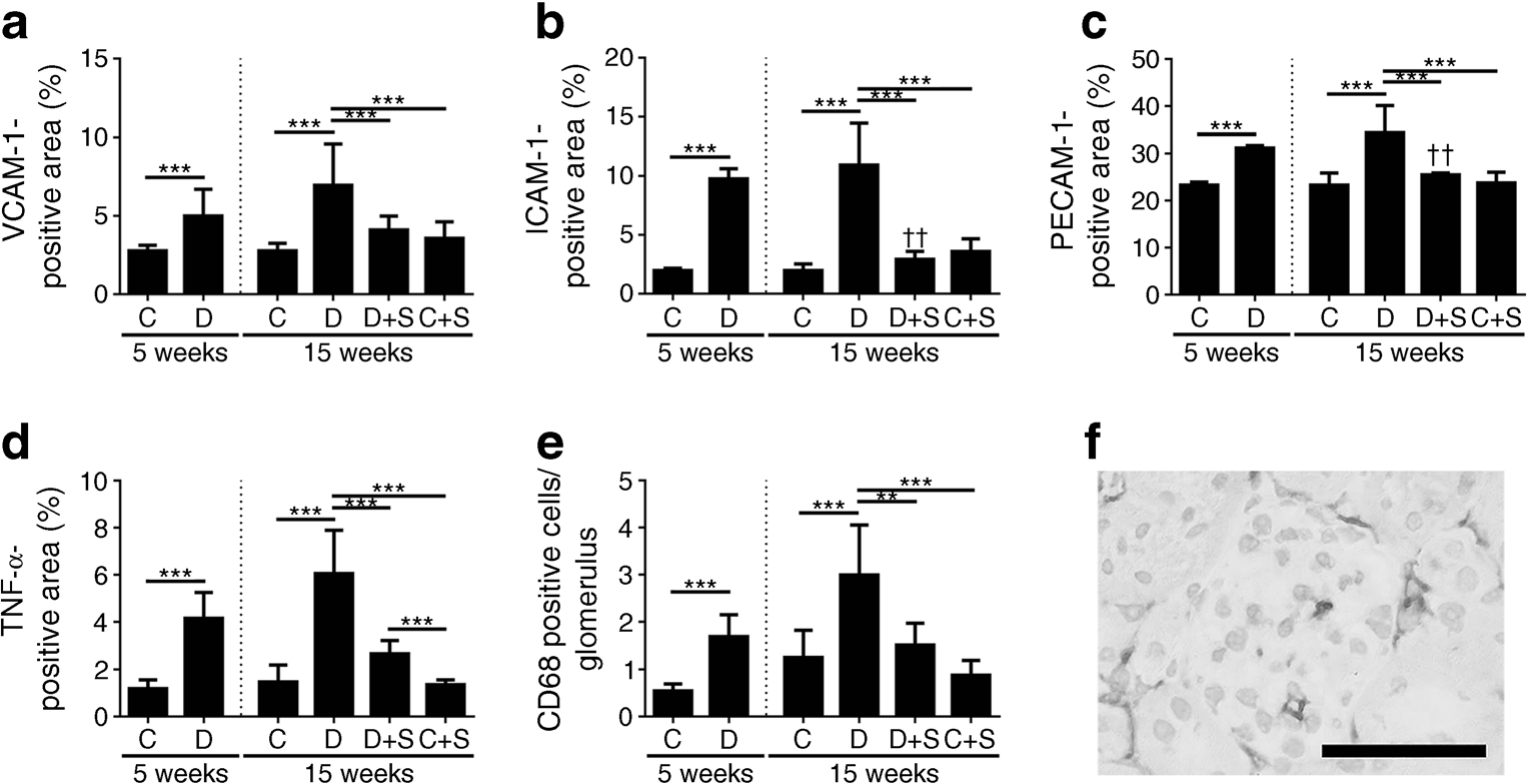
sFLT-1 reduced glomerular endothelial activation, the number of glomerular macrophages and glomerular inflammation in diabetic mice. Mice were injected with STZ to induce diabetes. In week 6, diabetic (D) and control (C) mice were transfected with a construct expressing *sFlt-1* (S). At 5 and 15 weeks, VCAM-1 (**a**), ICAM-1 (**b**), PECAM-1 (**c**), TNF-α (**d**) and the number of glomerular macrophages (**e**) were measured. ***p < 0.001, Student’s *t* test between groups at week 5. **p < 0.01 and ***p < 0.001, one-way ANOVA between groups at week 15. ^††^ *p* < 0.01 vs the corresponding diabetic mice at 5 weeks, one-way ANOVA after correcting for the time effect. Bars represent means ± SD. Number of animals: non-diabetic, non-transfected control mice at 5 and 15 weeks (*n* = 5 mice each); non-transfected diabetic mice at 5 and 15 weeks (*n* = 7 and *n* = 6, respectively); non-diabetic control mice transfected with *sFlt-1* (*n* = 10); and diabetic mice transfected with *sFlt-1* (*n* = 6). (**f**) Representative image of macrophages present in a glomerulus of a diabetic mouse at week 15 after staining for CD68. Scale bar, 50 μm

Next, we measured endothelial activation in diabetic and control mice at the 5-week time point. Compared with control mice, diabetic mice had increased glomerular endothelial activation, reflected by increased levels of VCAM-1, ICAM-1 and PECAM-1 (*p* < 0.001) (Fig. 3a–c). The diabetic mice also had increased levels of glomerular TNF-α (*p* < 0.001) (Fig. 3d) and increased numbers of glomerular macrophages (*p* < 0.001) (Fig. 3e, f) compared with control mice. At week 15, all three markers of glomerular endothelial cell activation remained increased in the diabetic mice compared with control (non-diabetic) mice (*p* < 0.001) (Fig. 3a–c). At week 15, the diabetic mice also had more infiltration of glomerular macrophages and increased levels of glomerular TNF-α compared with control mice (*p* < 0.001). Strikingly, transfection with *sFlt-1* significantly reduced all of these markers of glomerular endothelial activation and inflammation in the diabetic mice (*p* < 0.01); in most cases, the marker was reduced to control levels (Fig. 3a–e). Transfecting control (non-diabetic) mice with *sFlt-1* had no effect on any of the markers investigated (Fig. 3a–e). Compared with diabetic mice at week 5, *sFlt-1*-transfected diabetic mice at week 15 had significantly lower levels of ICAM-1 and PECAM-1 (*p* < 0.01) (Fig. 3b, c).

### sFLT-1 reduced VEGF-A-induced endothelial activation in a dose-dependent manner

**Fig. 4:**
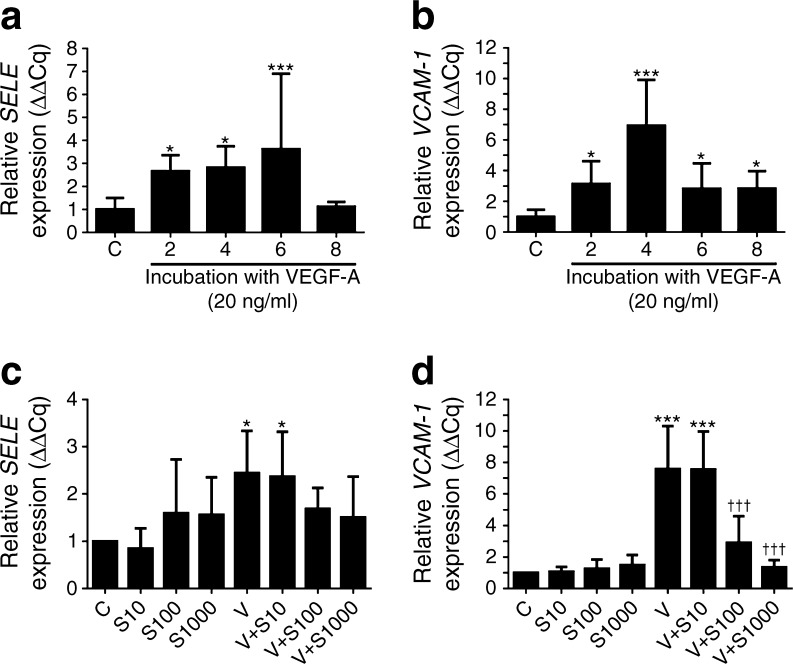
In vitro treatment with sFLT-1 reduced VEGF-A-induced endothelial activation in a dose-dependent manner. (**a**) *SELE* and (**b**) *VCAM-1* mRNA levels were measured in HUVECs incubated with 20 ng/ml VEGF-A for 2, 4, 6 or 8 h; each mRNA level is plotted relative to the respective level in untreated HUVECs. (**c**) *SELE* and (**d**) *VCAM-1* mRNA levels were measured in HUVECs incubated with 20 ng/ml VEGF-A for 4 h in the presence of 10, 100 or 1000 ng/ml sFLT-1 (S10, S100 and S1000, respectively). C, cells that were not treated with either VEGF-A or sFLT-1; V, cells stimulated with VEGF-A but not treated with sFLT-1. Each mRNA level is plotted relative to the respective level in untreated cells. Bars represent means ± SD. *p < 0.05 and ***p < 0.001 vs the respective untreated control group, one-way ANOVA. ^†††^ *p* < 0.001 vs the respective VEGF-A–stimulated group

Our data suggest that *sFlt-1* transfection in diabetic mice reduces kidney damage by reducing the glomerular infiltration of macrophages and by lowering the production of pro-inflammatory molecules such as TNF-α. Activation of endothelial cells is a key factor in this process, as it mediates the vascular adhesion of monocytes and their migration from the bloodstream into the tissue. Therefore, we investigated the in vitro effect of sFLT-1 on VEGF-A-induced endothelial activation. First, we measured the time course of VEGF-A-induced endothelial activation. Incubating HUVECs with 20 ng/ml VEGF-A-induced endothelial activation, reflected by significant increases in expression of the genes encoding E-selectin (*SELE*) and VCAM-1 (*VCAM-1*) compared with unstimulated HUVECs; the mRNA levels of *SELE* and *VCAM-1* peaked 6 and 4 h, respectively, after stimulation (Fig. 4a, b). In contrast, the expression of *ICAM-1* was not significantly affected by VEGF-A stimulation (data not shown).

Next, we investigated the effect of applying various concentrations of sFLT-1 on endothelial activation in HUVECs 4 h after stimulation with VEGF-A (Fig. 4c, d). We found that sFLT-1 significantly decreased the VEGF-A-induced upregulation of *VCAM-1* (*p* < 0.001) in a dose-dependent manner. sFLT-1 did not significantly affect the VEGF-induced upregulation of *SELE*. sFLT-1 had no effect on the mRNA levels of *SELE* or *VCAM-1* in unstimulated cells.

## Discussion

Here, we show that transfection with the VEGF-A inhibitor gene *sFlt-1* in mice with diabetic nephropathy reverses pre-existing kidney damage by normalising albumin:creatinine levels and mesangial matrix content. Furthermore, transfection with *sFlt-1* in diabetic mice also reduced endothelial activation (measured as VCAM-1, ICAM-1 and PECAM-1 protein levels), glomerular infiltration of macrophages and glomerular TNF-α protein levels. Finally, treating HUVECs with sFLT-1 decreased VEGF-A-induced endothelial activation in a dose-dependent manner. Taken together, these data suggest that treatment with sFLT-1 may be beneficial in individuals with diabetic nephropathy.

Animal models of diabetic nephropathy develop increased levels of glomerular VEGF-A [4, 5], and inhibiting VEGF-A in diabetic animal models can prevent the development of albuminuria, glomerular hypertrophy and podocyte loss [7–9, 23]. Consistent with these findings, podocyte-specific overexpression of *sFlt-1* has been reported to reduce mesangial expansion and glomerular basement membrane thickening in diabetic mice [24]. However, that study did not investigate the effect of systemic sFLT-1 treatment, which will likely be required to treat individuals with diabetes. In contrast, other studies have found that anti-VEGF-A treatment has no effect on early renal pathology [25], and that podocyte-specific deletion of *Vegfa* in diabetic mice causes increased proteinuria and kidney damage [21]. Moreover, although another study reported that treating diabetic mice with sFLT-1 decreased albuminuria, it did not reduce glomerular matrix deposition and led to an increase in tubular damage [26]. These conflicting results could be due to a variety of factors, including the time at which treatment is initiated, and the dose and/or type of anti-VEGF-A treatment used (e.g. an anti-VEGF-A antibody, a VEGFR2 inhibitor or sFLT-1). For example, using a construct in which domain 2 of FLT-1 is linked to human IgG1Fc may lead to increased inflammation due to binding to Fc receptors on macrophages (for review, see Guilliams et al [27]), increasing tubular damage [26] independent of sFLT-1. In contrast, we used a full-length sFLT-1 construct without an Fc tag. In addition, VEGF-A inhibitors such as native sFLT-1 may have beneficial functions in addition to binding VEGF-A. For example, sFLT-1 has been reported to bind to lipid microdomains in podocytes, thereby affecting the actin cytoskeleton and the function of the glomerular barrier [28]. Podocyte-specific deletion of *Flt-1* expression causes reorganisation of the cytoskeleton, leading to proteinuria and kidney damage; these effects are rescued by expressing a kinase-deficient mutant of *Flt-1*, suggesting that physiological levels of sFLT-1 are necessary for the proper structure and function of podocytes [28]. Therefore, with respect to kidney damage, treating individuals with sFLT-1 may provide improved outcomes compared with anti-VEGF-A antibodies and VEGFR2 inhibitors.

Importantly, the studies discussed above investigated the prevention—rather than the treatment—of diabetes-induced kidney damage, as therapy was initiated before the onset of kidney damage. Therefore, it is difficult to estimate the effects of such treatments in diabetic individuals who have already developed kidney damage. Fioretto et al reported that kidney lesions in diabetic individuals were reversed by normalising glycaemia levels as a result of pancreatic transplantation [29]. Therefore, we tested the effect of treating diabetic mice with the VEGF-A inhibitor sFLT-1 after the onset of kidney damage, including albuminuria and mesangial matrix accumulation. We found that even though transfection with *sFlt-1* did not normalise blood glucose levels in diabetic mice (ESM Fig. 2), kidney damage was reversed, as both albuminuria and mesangial matrix accumulation were reduced.

Several studies have reported that macrophages play a role in the development of diabetic nephropathy [10–12]. Moreover, VEGF-A plays a role in the migration of monocytes and macrophages [13] by binding the FLT-1 receptor on these cells [14, 30]. In addition, incubating endothelial cells with either glucose [17] or VEGF-A [16] results in endothelial activation, a key event in the adhesion and migration of monocytes from the circulation into the tissue. Consistent with this finding, both animals and people with diabetes have increased levels of endothelial activation [31–33]. Furthermore, we found that incubating HUVECs with VEGF-A increased endothelial activation, and that this effect was reversed by treating the cells with sFLT-1. We also found that transfection with *sFlt-1* normalised both the number of glomerular macrophages and the level of TNF-α in diabetic mice. Taken together, these findings suggest that the VEGF-A inhibitor sFLT-1 reduces endothelial activation and subsequent macrophage infiltration. Treatment with sFLT-1 has reported benefits in treating other diseases, including arthritis [34, 35], vascular disease [36, 37], sepsis [38] and psoriasis [39]; these clinical benefits are attributed primarily to reduced numbers of infiltrating macrophages and reduced inflammation. The current results indicate that sFLT-1 may be a valuable treatment for diabetic nephropathy, as well as other diseases in which inflammation plays an important role. Macrophages produce cytokines such as TNF-α and TGF-β, which increase the production of matrix proteins by mesangial cells [40]. Thus, reducing the number of glomerular macrophages using sFLT-1 might also reduce mesangial matrix expansion in diabetic nephropathy.

As reviewed by Deeds et al [41], techniques using STZ (such as dosage and administration) and consistency with respect to the resulting diabetes mellitus in small animal models have not been standardised. In our study, we used a moderate dosing regimen of three doses of 75 mg/kg STZ, for two reasons: (1) this regimen is less nephrotoxic than a single high dose; and (2) this regimen induces more diabetes-related histological damage compared with several low doses, which result in a relatively mild phenotype. In rodents, STZ can cause nephrotoxicity; however, Kraynak et al have reported that STZ-induced cellular and molecular damage resolves within 3 weeks [42]. This suggests that the albuminuria seen in our diabetic mice at 5 weeks was probably related to diabetes rather than to STZ. This is supported by the histological characteristics typical of diabetic nephropathy seen in these diabetic mice (i.e. mesangial matrix expansion and glomerular hypertrophy). Although some groups have reported albuminuria and histological lesions at this time point [43–45], other groups did not find albuminuria at this time point [31, 46]; this discrepancy may be due to differences in the dose and/or route of administration of STZ. It is important to note that although present, the albuminuria in our STZ-injected diabetic mice was not exceedingly high, and we suggest that the importance of albuminuria in C57BL/6 mice must be considered in combination with the presence (or absence) of histological findings.

Importantly, we found a small, but significant, decrease in podocyte numbers in *sFlt-1*-transfected diabetic mice; decreased numbers of podocytes have also been reported in pre-eclampsia, which is characterised by high circulating levels of sFLT-1 [47]. Despite this decrease in podocyte numbers, albuminuria was significantly reduced in *sFlt-1*-transfected diabetic mice. It is possible that the decrease in podocyte numbers in these *sFlt-1*-transfected mice was too small to functionally affect the filtration barrier. This notion is supported by previous reports that a substantial decrease in podocyte numbers is required for increased albuminuria [48, 49]. Nevertheless, we cannot exclude the possibility that longer treatment and/or higher levels of sFLT-1 expression could affect the glomerular filtration barrier. Thus, we hypothesise that sFLT-1 will likely have a beneficial effect in people with diabetes until the production of VEGF-A by podocytes drops below a certain threshold, given that decreased VEGF-A levels also result in kidney damage [21]. In this respect, it is important to note that both VEGF-A and sFLT-1 levels should be adjusted with care, as both increased and decreased levels of VEGF-A can lead to renal pathology [3, 50].

In conclusion, we report that normalising VEGF-A levels with sFLT-1 might be a viable approach for treating individuals with existing diabetic nephropathy by reducing endothelial activation, glomerular macrophage infiltration and glomerular inflammation, thereby reversing kidney damage.

## Electronic supplementary material

ESM Figs (PDF 250 kb)

## Abbreviations

DAB: Diaminobenzidine
FLT: Fms-related tyrosine kinase
HEK293: Human embryonic kidney 293
ICAM: Intercellular adhesion molecule
PECAM: Platelet/endothelial cell adhesion molecule
sFLT: Soluble fms-related tyrosine kinase
STZ: Streptozotocin
VCAM: Vascular cell adhesion molecule
VEGF: Vascular endothelial growth factor
VEGFR: VEGF receptor
VSV: Vesicular stomatitis virus
WT: Wilms tumour
