# Supplementary material for: The VEGF-A inhibitor sFLT-1 improves renal function by reducing endothelial activation and inflammation in a mouse model of type 1 diabetes
Source: Diabetologia. 2017 Jun 15;60(9):1813–21. doi: 10.1007/s00125-017-4322-3 (PMC5552850; doi:10.1007/s00125-017-4322-3)
Supplement: Supplementary file 1 — (PDF 250 kb) [file 125_2017_4322_MOESM1_ESM.pdf]

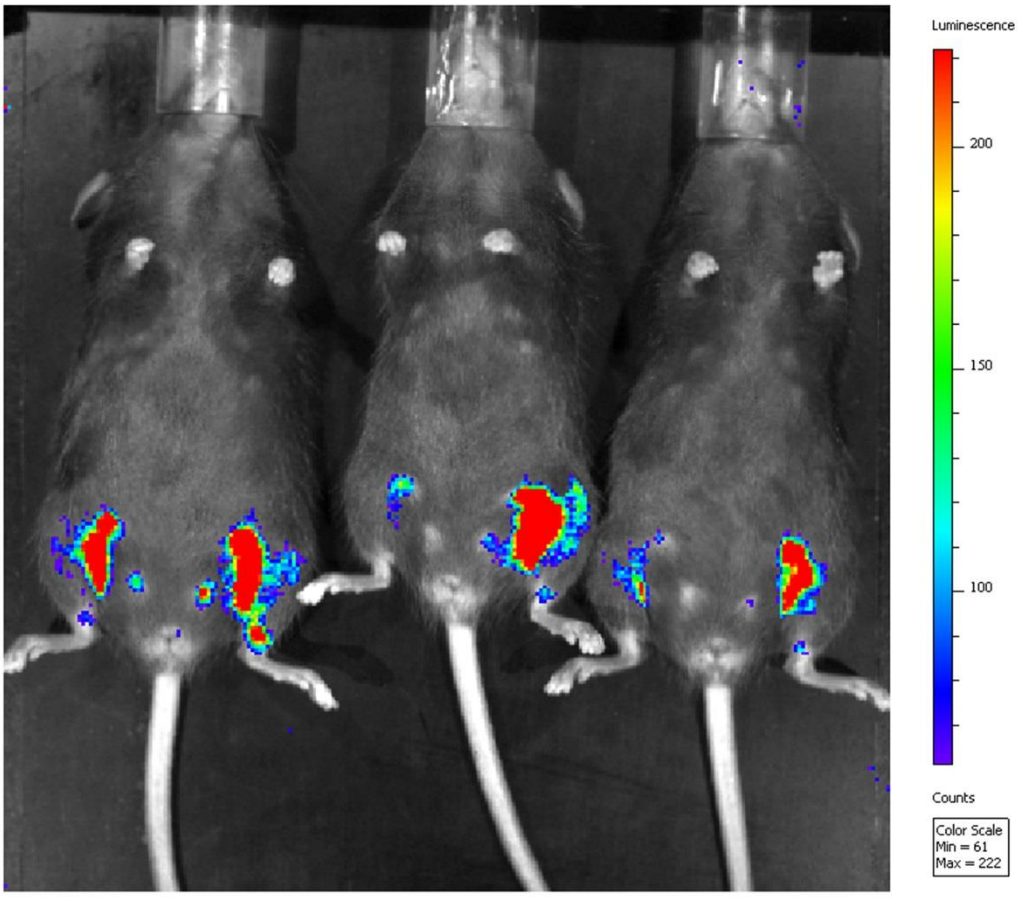

ESM Fig. 1 Transfection efficiency. The sFlt-1 and luciferase constructs were co-transfected bilaterally into both calf muscles. Following an i.p. injection of luciferin, luminescence was measured and is shown on an arbitrary pseudocolor scale; red color indicates high luminescence.

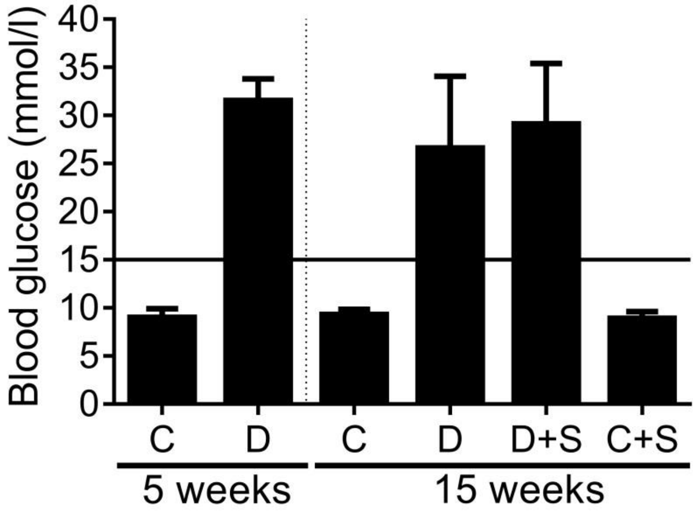

ESM Fig. 2 Blood glucose levels were measured in control (C) and diabetic mice (D) at 5 and 15 weeks. Where indicated, mice were transfected with *sFlt*-1 (S). The horizontal line at 15 mmol/L indicates the threshold used in this study to define diabetic mice. Transfection with *sFlt*-1 had no significant effect on blood glucose levels measured at week 15 in diabetic mice. Bars represent mean ±SD. Number of animals: non-diabetic, non-transfected control mice at 5 and 15 weeks (n=5 mice each); non-transfected diabetic mice at 5 and 15 weeks (n=7 and n=6, respectively); non-diabetic control mice transfected with *sFlt*-1 (n=10); and diabetic mice transfected with *sFlt*-1 (n=6).
